# Identification of potential biomarkers in the peripheral blood of neonates with bronchopulmonary dysplasia using WGCNA and machine learning algorithms

**DOI:** 10.1097/MD.0000000000037083

**Published:** 2024-01-26

**Authors:** Liyan Luo, Fei Luo, Chuyan Wu, Hong Zhang, Qiaozhi Jiang, Sixiang He, Weibi Li, Wenlong Zhang, Yurong Cheng, Pengcheng Yang, Zhenghu Li, Min Li, Yunlei Bao, Feng Jiang

**Affiliations:** a Department of Neonatology, Dali Bai Autonomous Prefecture Maternal and Child Health Care Hospital, Dali, China; b Department of Neonatology, Obstetrics and Gynecology Hospital of Fudan University, Shanghai, China; c Department of Rehabilitation Medicine, The First Affiliated Hospital of Nanjing Medical University, Nanjing, China.

**Keywords:** biomarker, bronchopulmonary dysplasia, diagnostic model, machine learning, weighted correlation network analysis

## Abstract

Bronchopulmonary dysplasia (BPD) is often seen as a pulmonary complication of extreme preterm birth, resulting in persistent respiratory symptoms and diminished lung function. Unfortunately, current diagnostic and treatment options for this condition are insufficient. Hence, this study aimed to identify potential biomarkers in the peripheral blood of neonates affected by BPD. The Gene Expression Omnibus provided the expression dataset GSE32472 for BPD. Initially, using this database, we identified differentially expressed genes (DEGs) in GSE32472. Subsequently, we conducted gene set enrichment analysis on the DEGs and employed weighted gene co-expression network analysis (WGCNA) to screen the most relevant modules for BPD. We then mapped the DEGs to the WGCNA module genes, resulting in a gene intersection. We conducted detailed functional enrichment analyses on these overlapping genes. To identify hub genes, we used 3 machine learning algorithms, including SVM-RFE, LASSO, and Random Forest. We constructed a diagnostic nomogram model for predicting BPD based on the hub genes. Additionally, we carried out transcription factor analysis to predict the regulatory mechanisms and identify drugs associated with these biomarkers. We used differential analysis to obtain 470 DEGs and conducted WGCNA analysis to identify 1351 significant genes. The intersection of these 2 approaches yielded 273 common genes. Using machine learning algorithms, we identified CYYR1, GALNT14, and OLAH as potential biomarkers for BPD. Moreover, we predicted flunisolide, budesonide, and beclomethasone as potential anti-BPD drugs. The genes CYYR1, GALNT14, and OLAH have the potential to serve as diagnostic biomarkers for BPD. This may prove beneficial in clinical diagnosis and prevention of BPD.

## 1. Introduction

Bronchopulmonary dysplasia (BPD) is a prevalent and complicated neonatal condition, with its incidence rates continuing to rise.^[1]^ In Europe and the US, approximately 28,000 and 18,000 babies, respectively, are affected by this condition every year.^[2,3]^ Premature infants who develop BPD often experience long-term respiratory and neurodevelopmental complications.^[4]^ The primary pathological alterations observed in individuals with BPD are simplified alveolar structures and pulmonary capillary dysplasia. In immature lungs, lung damage may result from a variety of factors, such as barotrauma, volutrauma, oxygen toxicity, fluid overload, genetic predispositions, infections, and inflammatory responses.^[5,6]^ Despite extensive research, the intricate etiology and complex pathogenesis of BPD pose significant challenges in identifying specific biomarkers for its accurate prediction and effective treatment.^[7]^

Rapid advancements in high-throughput microarray technologies and genome-wide sequencing techniques hold immense potential. They are pivotal not only for predicting, diagnosing, and treating human diseases but also for providing valuable insights into their pathology,^[8]^ while also providing valuable insights into their pathology. One typical application of these technologies is the discovery of biomarkers in diseases, both tumor-related and non-tumor-related. These biomarkers are instrumental in helping doctors predict patient prognosis and response to treatment.^[9,10]^ The combination of weighted gene co-expression network analysis (WGCNA) and random forest models has been instrumental in identifying key genes in various pathological processes, such as hormone secretion regulation and airway remodeling. This approach has been particularly helpful in understanding the progression of asthma.^[11]^ Furthermore, the integration of WGCNA with other machine learning methods, including the SVM-RFE model and LASSO analysis, has facilitated the identification of potential serum biomarkers, notably TMCC2, GYPA, and BPGM, in patients suffering from steroid-induced femoral head necrosis.^[12]^ However, it should be emphasized that the majority of bioinformatics research on BPD has not yet incorporated the use of WGCNA in conjunction with machine learning methods.

The challenge of obtaining human lung tissue has made peripheral blood a favored alternative. In recent years, the use of gene expression profiles from peripheral blood mononuclear cells (PBMCs) has gained prominence. Researchers utilize these profiles both to investigate lung disease pathogenesis and as a diagnostic tool.^[13]^ The primary aim of this research was to deepen our understanding of BPD pathogenesis and to enhance the methods available for its diagnosis and treatment. It is anticipated that the findings of this study will be instrumental in realizing these objectives.

## 2. Materials and methods

### 2.1. Data sources

This study aimed to identify potential biomarkers of BPD patients by analyzing RNA-seq data obtained from their blood samples and combining WGCNA with 3 machine learning algorithms. To start, the researchers performed differential expression gene (DEG) analysis and WGCNA hub module gene analysis from the Gene Expression Omnibus database. They then conducted functional analysis on the intersection of genes between these 2 analyses. Subsequently, the 3 aforementioned machine learning algorithms were employed to investigate the functions and regulatory mechanisms of these genes and identify potential biomarkers for BPD. To collect gene expression data for BPD, we utilized the GSE32472 dataset available in the Gene Expression Omnibus database. The research was carried out by the team at Polish American Children Hospital over the period from September 1, 2008, to November 30, 2010. The inclusion criteria for the study were as follows: a gestational age of <32 weeks at birth, a birth weight of 1500 g or less, and a requirement for respiratory assistance. All participants were born in nearby hospitals and subsequently transferred to the Polish American Children Hospital, a regional tertiary care facility. Contained within this dataset are microarray profiles from blood samples of newborns diagnosed with BPD. The gene expression levels in these samples were assessed around the 5th, 14th, and 28th days of life. For enhanced selection stability, we chose 100 blood samples from around the 28th day, a time frame that allows for a more precise BPD diagnosis. Jacek et.al provided the detailed methods regarding the experimental section of this study.^[14]^ These samples comprised 38 controls and 62 BPD cases. We utilized G*Power software to compute the Effect size (d) in assessing the statistical power associated with our sample size.^[15]^ After processing, the ggplot2 package in R was employed to normalize the data.

### 2.2. Functional enrichment analysis of DEGs in BPD

In this study, we used the “limma” and “GSEABase” packages in R (version 4.2.1) software to identify DEGs and perform gene set enrichment analysis (GSEA). The screening criteria used to identify DEGs between the BPD and control groups were a *P* value < .05 and |logFC| > 0.5.

### 2.3. Screening of target genes by WGCNA

To identify co-expression modules and construct unsigned co-expression networks, we utilized WGCNA in this study. To achieve this, we first checked the samples for missing values and clustered them. We then estimated a “soft” threshold power (β) based on the criteria of scale-free topology to construct a biologically relevant scale-free network. Furthermore, we generated a topological overlap matrix using an adjacency matrix and employed a dynamic tree-cutting algorithm to identify gene modules. To further analyze the modules, we computed both gene significance and module membership, and linked the modules with clinical features, visualizing the network of significant genes. Ultimately, we identified potential gene targets for BPD by determining the overlapped genes between DEGs and significant module genes derived from WGCNA.

### 2.4. Functional enrichment analysis

To analyze potential targets, we conducted GO, DO, and KEGG enrichment analyses in this study. To achieve this, we utilized the “clusterProlifer” package and set a *P* value < .05 as the filtering criterion for functional analysis. First, we conducted GO analysis to identify biological processes, cellular components, and molecular functions related to hub genes. Additionally, we performed DO analysis to identify diseases that frequently involve the aforementioned genes. Finally, we conducted KEGG enrichment analysis to identify relevant pathways.

### 2.5. Protein-protein interaction (PPI) network construction

In this study, we utilized the STRING database to construct an interaction network of potential targets. We set the minimum required interaction score to medium confidence (0.4) to ensure the reliability of the interactions included in the network. The results were visualized using Cytoscape software. The genes that were included in the interaction network are involved in the pathological process of BPD, and were subsequently selected for biomarker screening in BPD patients.

### 2.6. Biomarker screening in BPD neonates by machine learning algorithms

In this study, we employed 3 distinct machine learning algorithms. Initially, the SVM-RFE analysis was executed using the “e1071” package. Following that, we conducted random forest analysis utilizing the “randomForest” package. Additionally, LASSO regression analysis was performed using the “glmnet” package. Genes identified as an overlap from the outcomes of these 3 machine learning algorithms were regarded as potential biomarkers for individuals with BPD. To further this, we constructed a nomogram centered on these hub genes using the “rms” package, aiming to estimate the occurrence of BPD based on these potential biomarkers.

### 2.7. Correlation and GSEA of potential biomarkers

The “corrplot” package was used to conduct correlation analysis on the expression of potential biomarkers. After that, GSEA was performed on the potential biomarkers to gain deeper insights into their functions.

### 2.8. Potential biomarkers regulatory mechanisms

To gain insights into the role of potential biomarkers in the development and progression of BPD, we conducted an enrichment analysis to identify transcription factors (TFs) associated with the potential biomarkers using the NetworkAnalyst platform. Furthermore, we predicted miRNAs that may regulate the expression of the identified biomarkers.

### 2.9. Potential drug screening

In this study, our aim was to address the limited efficacy of currently available drugs for BPD by identifying potential biomarkers and predicting drugs that target them. We searched the Drug Signatures Database (DSiDB) in the Enrichr website to predict drugs that target the identified biomarkers based on previous research. We also utilized the PubChem database to screen and visualize the chemical structures of the corresponding drugs.

### 2.10. Nomogram model construction

To predict the incidence of BPD, we utilized the rms package to construct a diagnostic nomogram model. The model predictive performance was assessed by a calibration curve, and its practical utility was evaluated using decision curve analysis. We also employed the pROC package to generate ROC curves and calculate the area under the curve to evaluate the diagnostic capability of hub genes.

## 3. Results

### 3.1. Characteristics of the study population

The research group included 100 participants, where 62 were identified with BPD at 28 days after birth. Demographic details of the group, categorized by the occurrence of BPD, are outlined in Table 1. It was noted that babies with BPD typically were born at earlier gestational age (26.4 weeks compared to 29.7 weeks, statistically significant with *P* < .01) and had lower birth weights (average 882 g vs 1235 g, with a significance of *P* < .01). Among those diagnosed with BPD, 14 out of 62 were also diagnosed with severe BPD by the 36th week of gestational age. The average birth weight and gestational age for this subgroup were 725 g and 25.3 weeks, respectively.

**Table 1:** Patient characteristics.

Variable	Overall (n = 100)	No BPD (n = 38)	BPD (n = 62)	Mild/moderate BPD (n = 48)	Severe BPD (n = 14)	*P* value
Gestational age, wk	27.6 (22.0, 32.0)	29.7 (26.0, 32.0)	26.4 (22.0, 31.0)	26.7 (22.0, 31.0)	25.3 (22.0, 31.0)	<.01
Birth weight, g	1016 (550, 1500)	1235 (750, 1500)	882 (550, 1440)	928 (550, 1440)	725 (550, 1300)	<.01
Female	44 (44%)	21 (55.3%)	23 (37.1%)	18 (37.5%)	5 (35.7%)	.08
Periventricular Leukomalacia	16 (16%)	2 (5.3%)	14 (22.6%)	10 (20.8%)	4 (28.6%)	.02
Retinopathy of prematurity	46 (46%)	3 (7.9%)	43 (69.4%)	30 (62.5%)	13 (92.9%)	<.01

Values are median with interquartile range for continuous data and number with percentage for categorical variables. Statistical analysis included Wilcoxon rank-sum test and Chi-square for continuous and categorical data, respectively. *P* value compares No BPD vs BPD groups.

BPD = bronchopulmonary dysplasia.

### 3.2. Identification of DEGs in BPD patients

**Figure 1.**
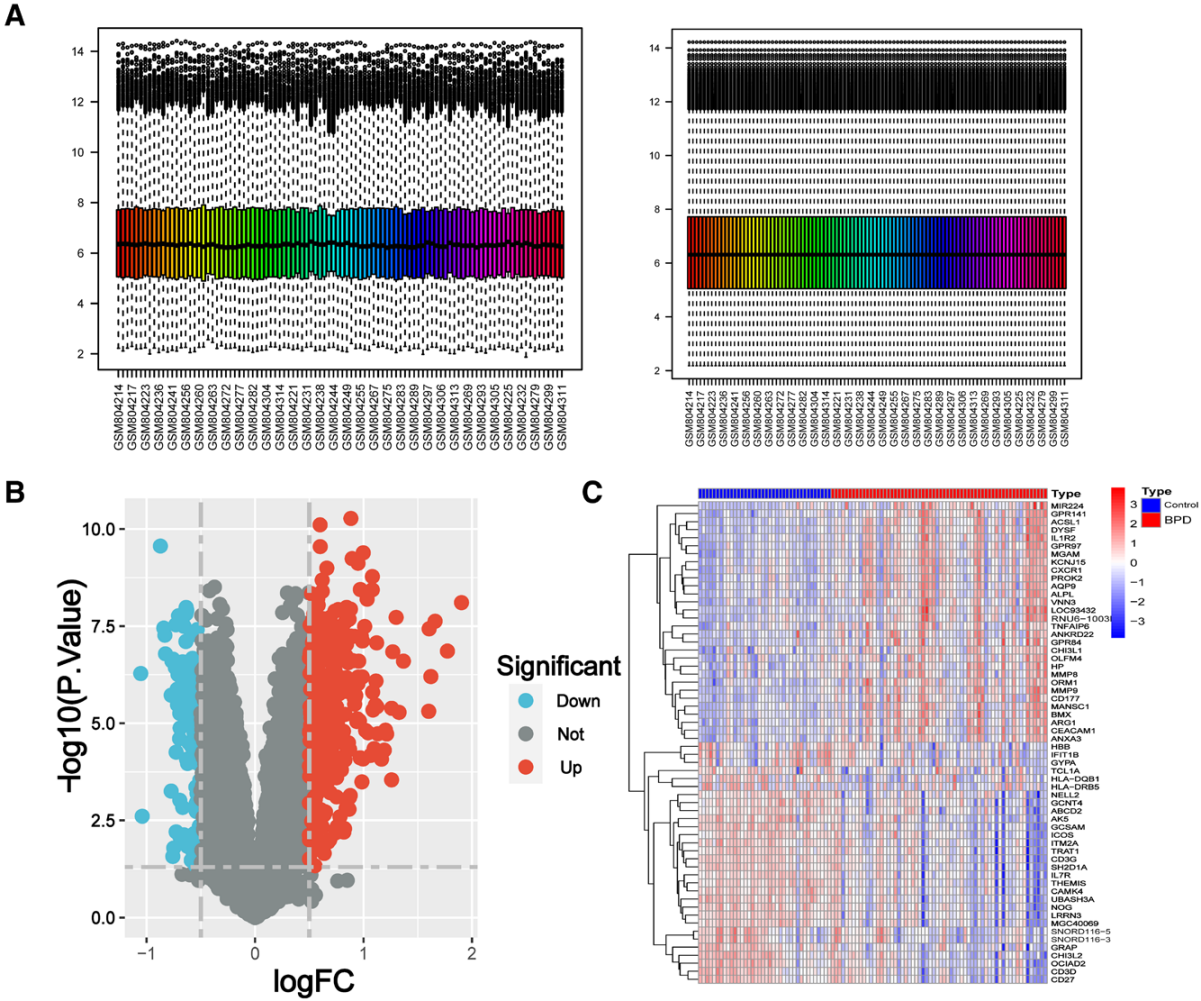
Identification of DEGs of BPD. (A) The normalization process was applied to the samples. (B) The volcano plot depicts the expression patterns of DEGs, where red denotes genes upregulated in BPD group and blue represents genes upregulated in the control group. (C) The expression of the top 30 DEGs in the sample is presented in the heat map. BPD = bronchopulmonary dysplasia, DEG = differential expression gene.

**Figure 2.**
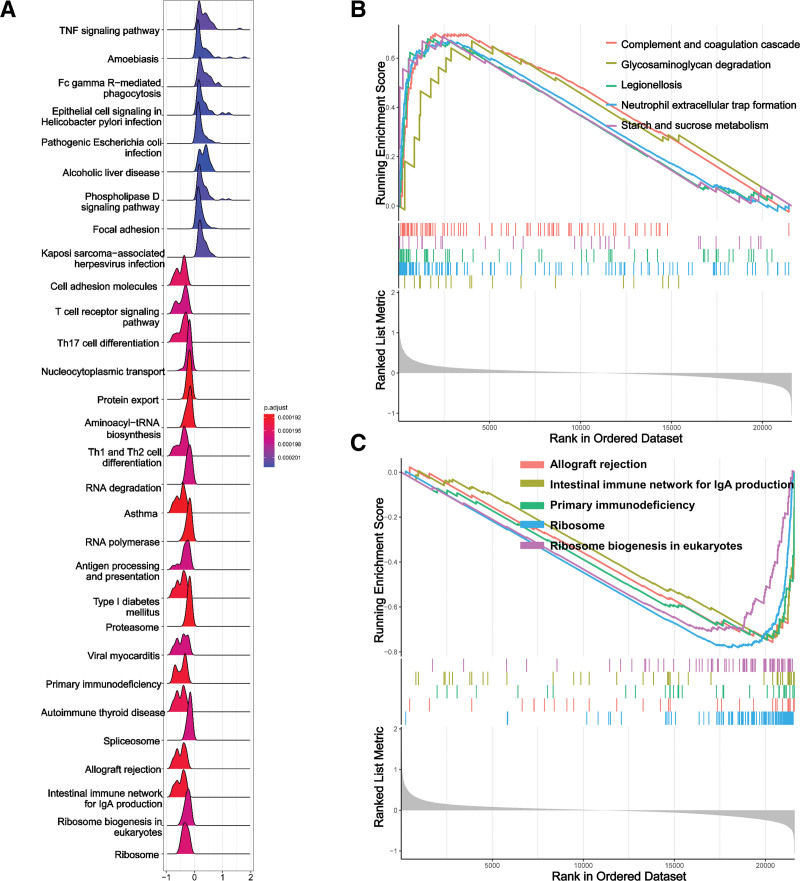
GSEA analysis for DEGs. (A) A ridgeline plot was utilized to illustrate the results of GSEA analysis. (B) The top 5 enrichment terms for upregulated DEGs are presented. (C) The top 5 enrichment terms for downregulated DEGs are listed. DEG = differential expression gene, GSEA = gene set enrichment analysis.

The peripheral serum samples were standardized, resulting in the identification of 470 DEGs in patients with BPD (Fig. 1A). Among the DEGs identified, 334 were up-regulated and 136 were down-regulated, as shown in Figure 1B. Figure 1C displays the top 30 genes with the greatest differences between the BPD and control groups. Subsequently, GSEA of the 470 DEGs revealed that upregulated genes were significantly enriched in complement and coagulation cascades, glycosaminoglycan degradation, legionellosis, neutrophil extracellular trap formation, starch, and sucrose metabolism (Fig. 2A and B). On the other hand, downregulated genes were significantly enriched in allograft rejection, primary immunodeficiency, ribosome, and ribosome biogenesis in eukaryotes (Fig. 2A and C).

### 3.3. Overlapped genes between BPD-related module genes with DEGs

**Figure 3.**
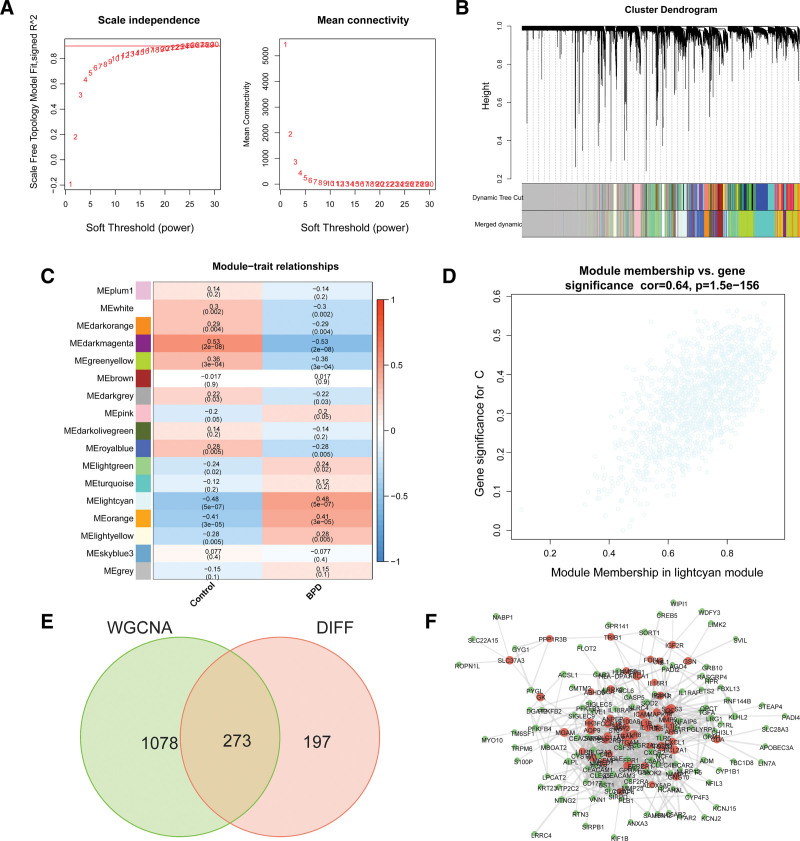
The identification of crucial modules was accomplished through WGCNA. (A) The scale-free fit index and mean connectivity were calculated for various soft-thresholding powers. (B) Topological overlap dissimilarity was used to aggregate DEG clusters. (C) The module-feature correlations are presented. (D) A scatter plot of the lightcyan module is depicted. (E) The Venn diagram demonstrates the genes that overlap. (F) PPI network of overlapped genes. DEG = differential expression gene, PPI = protein-protein interaction, WGCNA = weighted gene co-expression network analysis.

A soft threshold of 22 was used to construct a scale-free network, as shown in Figure 3A. We calculated the module eigengenes, which represent the total gene expression level of each module and are grouped based on their association. A total of 17 modules were identified, as shown in Figure 3B. The lightcyan module demonstrated the highest correlation with BPD (cor = 0.64, *P* = 1.5 × 10^156^), and the 1351 BPD-associated genes in this module were selected for further analysis, as illustrated in Figure 3C and D. Among them, 273 genes overlapped with the DEGs and were identified as potential targets for further analysis, as shown in Figure 3E. Furthermore, we constructed a PPI network for these 273 overlapping genes using the STRING database (Fig. 3F).

### 3.4. Functional enrichment analysis

**Figure 4.**
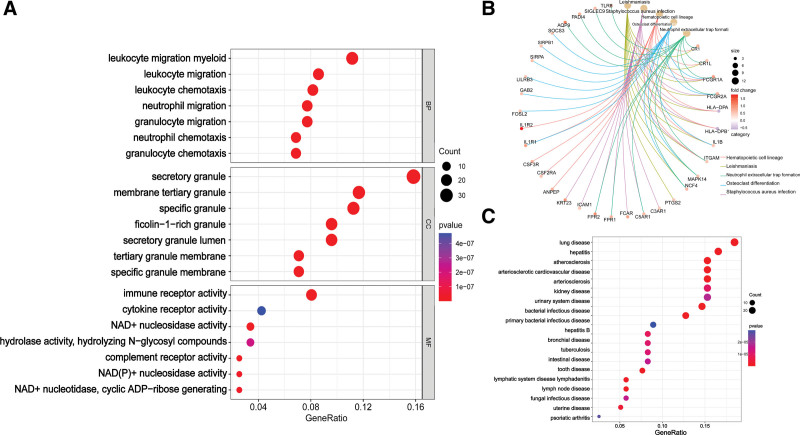
Functional overlapped genes functional enrichment. (A) GO. (B) KEGG. (C) DO.

The top GO terms identified by the GO analysis were presented in Figure 4A. The overlapping genes between the BPD-related module genes and DEGs were significantly enriched in processes such as leukocyte migration, myeloid leukocyte migration, secretory granule membrane, tertiary granule, immune receptor activity, and cytokine receptor activity. The KEGG analysis showed that the overlapping genes were enriched in pathways related to hematopoietic cell lineage, leishmaniasis, and neutrophil extracellular trap formation (Fig. 4B). The DEGs were found to be enriched in diseases such as lung disease, hepatitis, and atherosclerosis, as determined by DO analysis (Fig. 4C).

### 3.5. Hub gene identification

**Figure 5.**
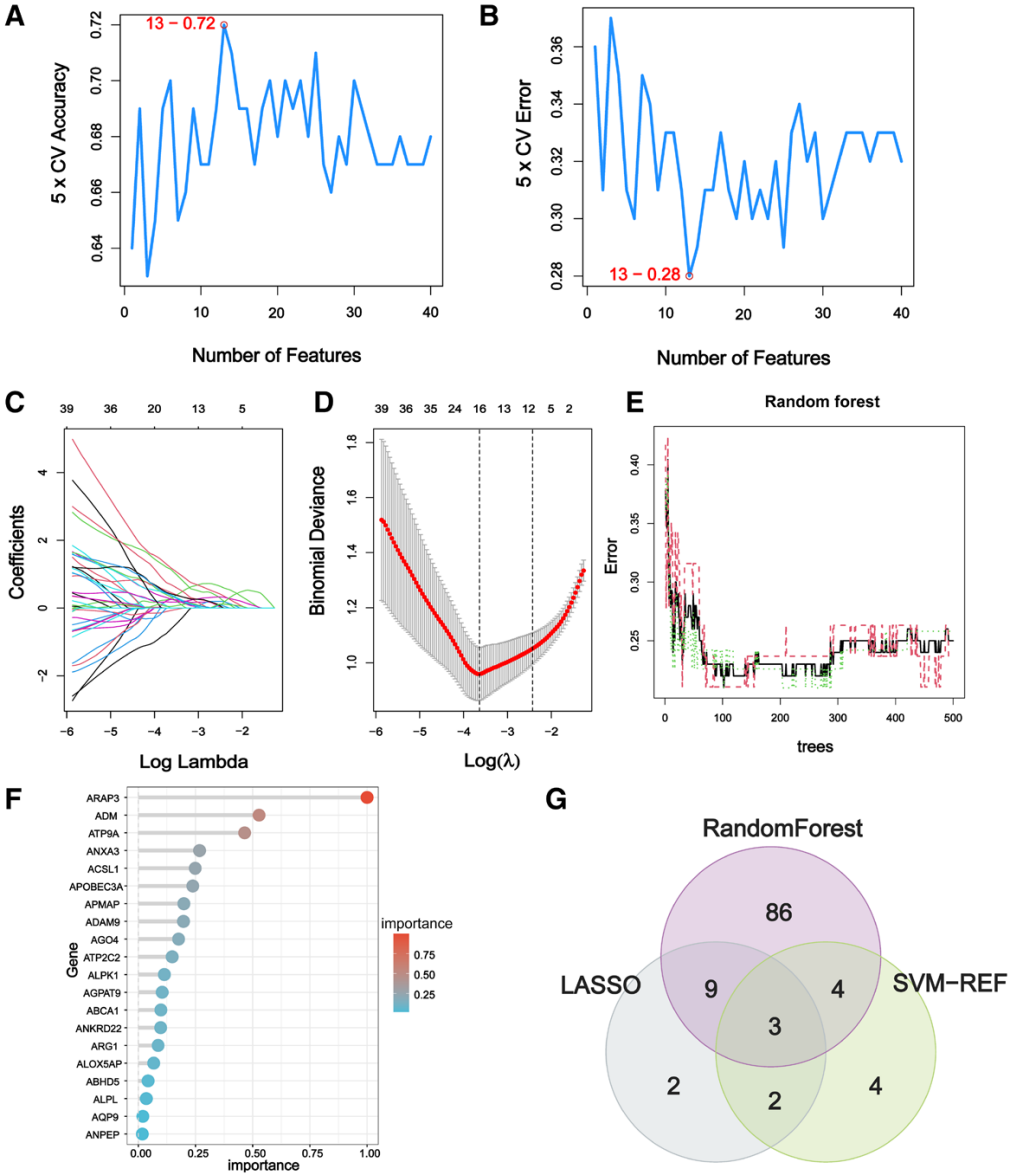
The screening for potential biomarkers was conducted using a machine learning approach. (A) SVM-RFE analysis identified 13 gene signatures with an accuracy of 0.72. (B) The error rate was calculated to be 0.28. (C) Cross-validation was conducted to determine the optimal tuning parameter, log (Lambda), for LASSO regression analysis. (D) The coefficient profiles of the candidate genes obtained from LASSO regression analysis are presented. (E) The prediction accuracy of the RF model was determined. (F) The gene importance scores of the RF model are presented. (G) The Venn diagram illustrates that CYYR1, GALNT14, and OLAH were identified as 3 hub genes shared by the 3 machine learning algorithms.

**Figure 6.**
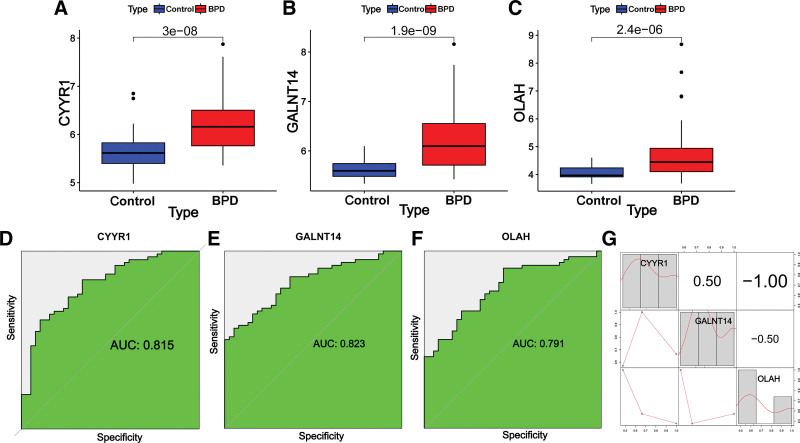
The expression and diagnostic values of hub genes in BPD. (A–C) The expression levels of 3 hub genes were compared between the BPD and control groups. (D–F) ROC curves and AUC statistics were used to assess the diagnostic performance of the hub genes in predicting the incidence of BPD. (G) Correlation between hub genes. AUC = area under the curve, BPD = bronchopulmonary dysplasia.

In order to identify gene signatures, we utilized SVM-RFE, RF, and LASSO on the 273 candidate genes. From the SVM method, we identified a 13-gene signature with a precision of 0.72 (Fig. 5A and B). LASSO regression analysis yielded 16 gene signatures (Fig. 5C and D), while the random forest method identified 102 genes with importance scores (Fig. 5E and F). We then obtained 3 hub genes, CYYR1, GALNT14, and OLAH, by overlapping the genes from the 3 methods (Fig. 5G). Notably, all 3 hub genes were significantly increased in BPD samples compared to controls (Fig. 6A–C), and the area under the curve values for these genes were >0.8, indicating good diagnostic efficacy (Fig. 6D–F). Furthermore, correlation analysis revealed that the 3 genes were robustly correlated with each other (Fig. 6G), making them promising candidates for further investigation.

### 3.6. Gene set enrichment of the hub genes

**Figure 7.**
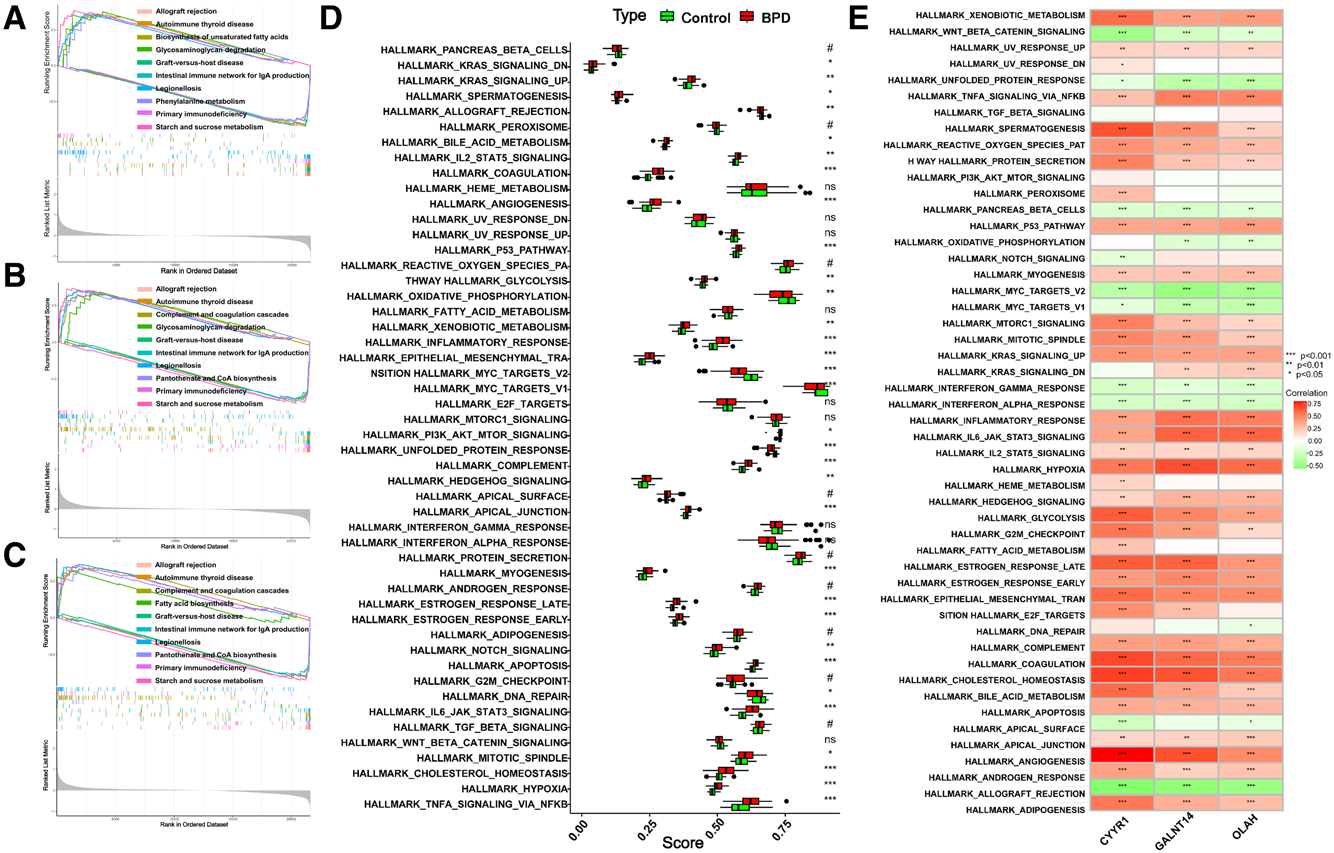
GSEA analysis of the 3 hub genes. (A–C) ssGSEA analysis of CYYR1, GALNT14, and OLAH. (D) hallmark pathways scores in BPD and control groups. (E) correlation between hallmark pathways scores and 3 hub genes. BPD = bronchopulmonary dysplasia, GSEA = gene set enrichment analysis.

To gain more insights into the potential functions of CYYR1, GALNT14, and OLAH, we performed GSEA. The findings suggest that the genes categorized as having high expression levels among the 3 hub genes were notably enriched in allograft rejection and autoimmune thyroid disease, as well as biosynthesis of unsaturated fatty acids, as illustrated in Figure 7A–C. Furthermore, we calculated hallmark pathway scores for the GSE32472 samples using the ssGSEA method. As shown in Figure 7D, coagulation, angiogenesis, the P53 pathway, and inflammatory response were significantly enriched in the BPD groups. Additionally, samples with upregulated expression of CYYR1, GALNT14, and OLAH exhibited higher enrichment scores in the pathways of TNF-α signaling via NFκB, spermatogenesis, the reactive oxygen species pathway, protein secretion, and the MTORC1 signaling pathway (Fig. 7E).

### 3.7. Diagnostic model construction

**Figure 8.**
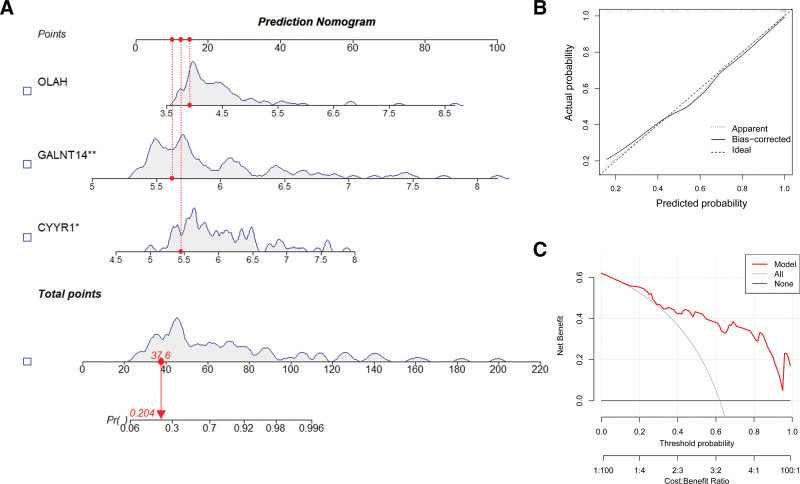
A nomogram model was developed to aid in the diagnosis of BPD. (A) A nomogram was created for predicting the risk of BPD. (B) The diagnostic performance of the nomogram model was assessed using calibration curves. (C) The practical effectiveness of the nomogram was evaluated using DCA curves. BPD = bronchopulmonary dysplasia, DCA = decision curve analysis.

A nomogram was developed for BPD diagnosis using CYYR1, GALNT14, and OLAH, as illustrated in Figure 8A. The calibration curve demonstrated that the nomogram model had excellent predictive ability with minimal variation between observed and predicted risk, as indicated in Figure 8B. Furthermore, decision curve analysis indicated that the hub gene nomograms had a substantial net benefit in predicting BPD risk at a risk threshold of 0.1 to 1.0, as shown in Figure 8C.

### 3.8. Regulatory mechanisms of potential biomarkers

**Figure 9.**
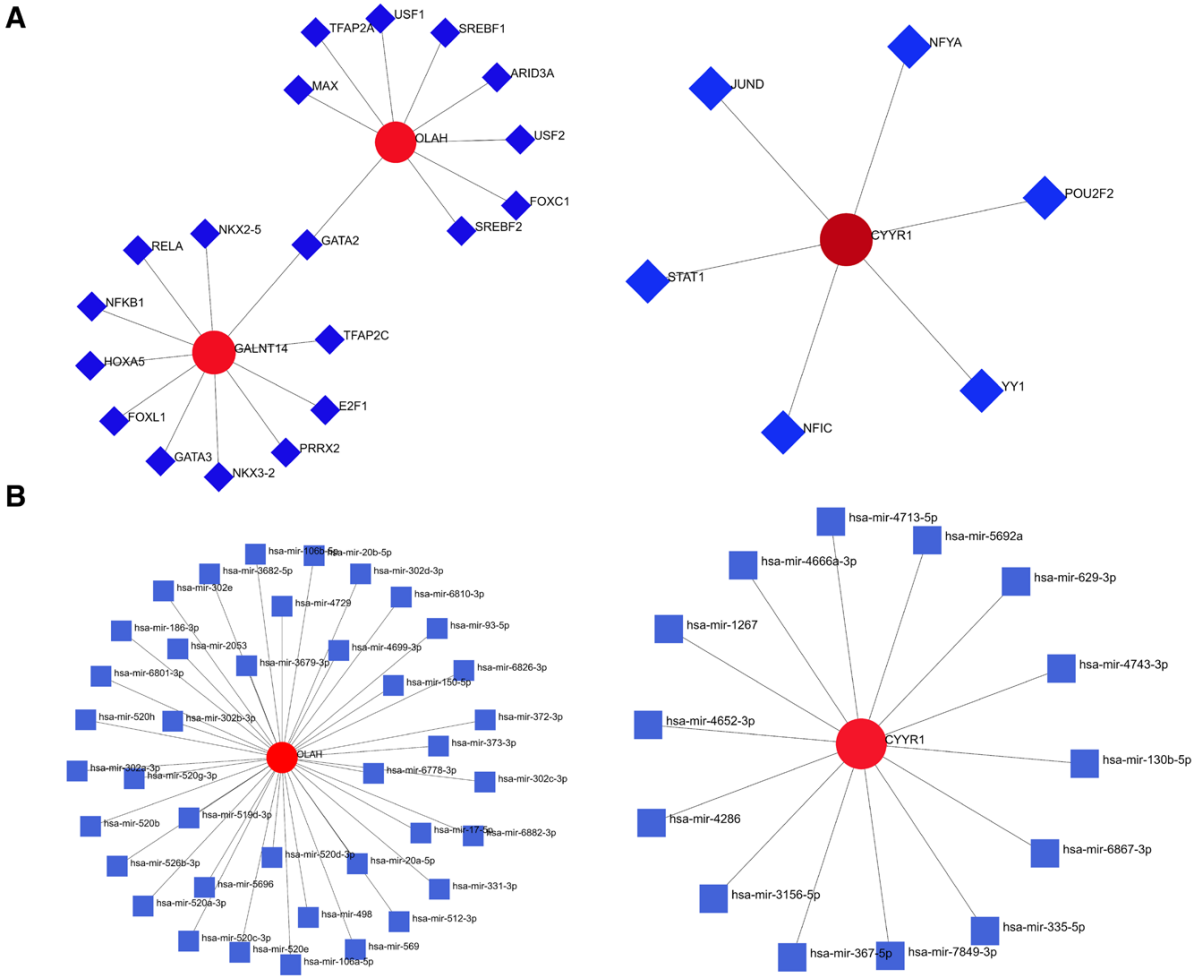
Regulatory mechanisms of potential biomarkers. (A) TF-potential biomarker network. (B) miRNA-potential network. TF = transcription factor.

In order to investigate the potential regulatory mechanisms of CYYR1, GALNT14, and OLAH, we used computational methods to predict their TFs. Subsequently, we constructed a network depicting the potential interactions between these TFs and the identified biomarker genes, as shown in Figure 9A. Based on our analysis, GALNT14 may be regulated by TFAP2C, E2F1, and FOXL1, while OLAH may be regulated by TFAP2A, ARID3A, and FOXC1. Moreover, our analysis revealed that CYYR1 may be regulated by STAT1, JUND, and NFIC. Next, we constructed a miRNA-potential biomarker network to identify miRNAs that potentially regulate these hub genes, as shown in Figure 9B. Our results suggest that hsa-mir-150-5p, hsa-mir-512-3p, and hsa-mir-335-5p may play important regulatory roles in these genes.

### 3.9. Prediction of therapeutic drugs

**Figure 10.**
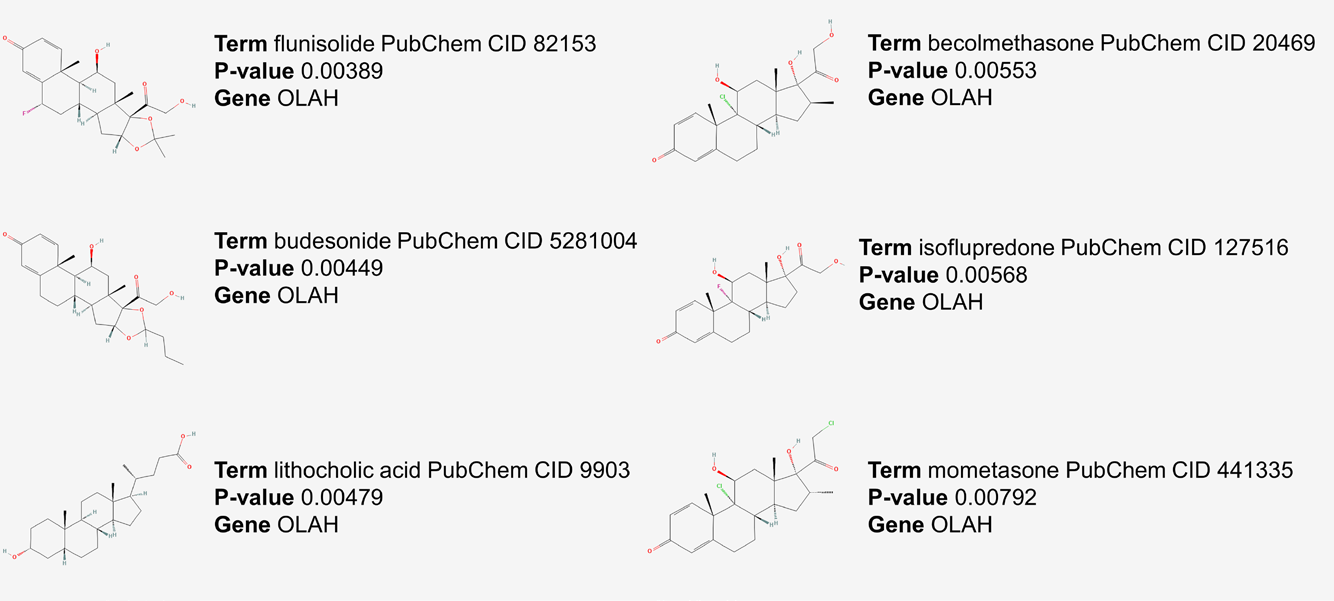
Drug prediction results.

Based on the 3 potential biomarkers of BPD patients, we used the DSigDB database to predict drugs with relevant effects. Six drugs with potential biomarker interactions were considered based on their *P* values in the prediction results, including flunisolide, budesonide, lithocholic acid, beclomethasone, isoflupredone, and mometasone, as shown in Figure 10.

## 4. Discussion

BPD remains a leading cause of mortality and long-term morbidity, despite advancements in neonatal intensive care medicine.^[16]^ Key features of BPD include reduced alveolar counts, increased alveolar capacity, and associated vascular complications.^[17]^ Alongside impacting lung functionality, these pathological aspects could result in enduring physical and cognitive developmental complications, potentially leading to fatality.^[18]^ A primary contributor to BPD in preterm infants is the buildup of oxidative stress.^[19]^ Unfortunately, the mechanisms underlying BPD pathogenesis remain poorly understood, underscoring the critical need to uncover these pathogenic mechanisms. The rapid development of gene chip technology has facilitated extensive genomic analysis in disease research. This technology presents a novel approach for identifying new therapeutic targets and extensively exploring biomarkers for BPD. To the best of our knowledge, this study represents the first attempt to combine differential analysis and WGCNA for the identification of hub genes, employing multiple machine learning algorithms in biomarker detection. The identified biomarkers have the potential to revolutionize disease diagnosis, inform the selection of therapeutic strategies, and predict responses to treatment.

In this study, we first identified 470 DEGs of BPD neonates. Subsequently, GSEA functional analysis of these genes revealed their involvement in pathways such as complement and coagulation cascades, glycosaminoglycan degradation, legionellosis, neutrophil extracellular trap formation, starch and sucrose metabolism, ribosome, and ribosome biogenesis in eukaryotes. These pathways, such as neutrophil extracellular trap formation and glycosaminoglycan degradation have been widely reported to play an important role in the development of BPD and immature immune systems in premature infants.^[20–24]^

Initially, 1351 genes were obtained from WGCNA, and then, after intersection analysis, 273 intersection genes were identified. GO analysis revealed that these genes were associated with leukocyte migration, myeloid leukocyte migration, neutrophil migration, secretory granule membrane, immune receptor activity, and cytokine receptor activity. These genes were also enriched in KEGG pathways such as hematopoietic cell lineage, neutrophil extracellular trap formation, and osteoclast differentiation. Previous studies have reported on the potential role of leukocyte adhesion and cytokine receptors in BPD pathogenesis.^[25,26]^ Additionally, DO enrichment analysis identified key targets primarily involved in lung disease, hepatitis, atherosclerosis, and arteriosclerotic cardiovascular disease, which may have a relationship with BPD that warrants further investigation.

Furthermore, we investigated the association among 273 target genes by constructing PPI networks, ultimately leading to the discovery of potential diagnostic biomarkers for BPD in peripheral blood: CYYR1, GALNT14, and OLAH. Several biomarkers have undergone examination for early BPD prediction.^[3,27–29]^ These markers hold promise in promptly diagnosing BPD, allowing for timely intervention to mitigate the occurrence of BPD and subsequent long-term cardiorespiratory complications. Wang et al suggest a valuable strategy for predicting BPD by combining clinical data, molecular biomarkers, and echocardiogram measurements. Among the factors considered, the tricuspid regurgitation jet, N-terminal-pro-B-brain natriuretic peptide, ventilator-associated pneumonia, days with FiO2 ≧ 40%, red blood cell volume, and the duration of infants receiving total enteral milk (120 Kcal/kg/day) for ≥ 24 days after birth emerge as the most viable predictors for BPD risk.^[30]^ In contrast, Gaertner et al utilize electrical impedance tomography to assess both regional ventilation distribution and overall lung aeration. Their findings emphasize that electrical impedance tomography markers evaluating aeration at 30 minutes after birth accurately forecast the need for oxygen supplementation at 28 days of age. However, these markers do not reliably predict the necessity for intubation or the likelihood of developing BPD.^[31]^ In our research, CYYR1, also referred to as cysteine and tyrosine-rich protein 1, is a newly discovered gene located on human chromosome 21 with an unclear protein function.^[32]^ Intriguingly, high expression of human CYYR1 was observed in cells belonging to the diffuse neuroendocrine system, which may potentially indicate a connection to neuroendocrine tumors.^[33]^ On the other hand, GALNT14 is a member of the GALNT family responsible for initiating O-glycosylation by adding GalNAc to a mucin-type protein serine or threonine residue.^[34]^ Numerous studies have investigated the diverse roles of GALNT14 in different types of cancer, including modulation of apoptotic signaling, tissue invasiveness, and migratory properties. Additionally, it has been found that the expression of GALNT14 can regulate multidrug resistance in breast cancer cells, function as a prognostic marker for neuroblastoma and non-small cell lung cancer, and predict response to Apo2L/TRAIL-based cancer therapies.^[35–38]^ Studies have shown that OLAH is upregulated in mammary gland epithelial cells and bone marrow-derived mononuclear cells of individuals with rheumatoid arthritis, and it plays a critical role in the synthesis of broad-distribution fatty acids present in breast milk. Moreover, OLAH expression in human placental tissue is higher than in other species, indicating its unique function in the human placenta. Additionally, we performed ssGSEA analysis to investigate the correlation between these 3 potential biomarkers and hallmark, and obtained clues. We also explored the regulatory mechanisms of these biomarkers. TF prediction revealed that GALNT14 may be regulated by TFAP2C, E2F1, FOXL1, and other factors, while CYYR1 may be regulated by JUND, STAT1, and other factors. The predicted regulatory relationships by miRNAs suggest that OLAH may be regulated by hsa-mir-150-5p and hsa-mir-17-5p, while CYYR1 may be regulated by hsa-mir-629-3p and hsa-mir-130b-5p. Despite this, the exact mechanisms of their actions are not well understood and need further investigation in future studies. Additionally, we have identified several drugs that could potentially be used for treatment, including flunisolide, budesonide, lithocholic acid, beclomethasone, isoflupredone, and mometasone. Some of these drugs are glucocorticoids, but their effectiveness in treating neonatal BPD requires more extensive clinical research in the future.

This study has several limitations that need to be acknowledged. Firstly, the availability of transcriptome datasets in peripheral blood of BPD is limited, which may have impacted our analysis. Secondly, while we have identified potential biomarkers, their expression characteristics require further validation through additional literature support and clinical trials in animal models and human subjects.

## 5. Conclusions

In brief, our study has pinpointed 273 significant targets prevalent in BPD patients. Employing 3 distinct machine learning algorithms, namely, CYYR1, GALNT14, and OLAH, we’ve established their potential diagnostic relevance as peripheral blood biomarkers for BPD. Delving deeper into the mechanisms underlying these genes could offer novel perspectives for preventing, diagnosing, and treating BPD. However, a comprehensive understanding of the precise action mechanisms of these 3 genes in the onset and advancement of BPD necessitates further investigation.

## Author contributions

**Conceptualization:** Liyan Luo.

**Data curation:** Liyan Luo, Sixiang He.

**Formal analysis:** Fei Luo, Pengcheng Yang.

**Investigation:** Fei Luo, Weibi Li.

**Methodology:** Weibi Li, Yurong Cheng.

**Project administration:** Chuyan Wu.

**Resources:** Chuyan Wu, Wenlong Zhang.

**Software:** Hong Zhang, Zhenghu Li.

**Supervision:** Hong Zhang, Min Li, Feng Jiang.

**Validation:** Qiaozhi Jiang.

**Visualization:** Qiaozhi Jiang, Yunlei Bao.

**Writing – review & editing:** Feng Jiang.
